# Advanced ImageJ Analysis in Degenerative Acquired Vitelliform Lesions Using Techniques Based on Optical Coherence Tomography

**DOI:** 10.3390/biomedicines11051382

**Published:** 2023-05-06

**Authors:** Ioana Damian, George-Adrian Muntean, Larisa-Bianca Galea-Holhoș, Simona-Delia Nicoară

**Affiliations:** 1 Department of Ophthalmology, “Iuliu Hatieganu” University of Medicine and Pharmacy, 8 Victor Babeș Street, 400012 Cluj-Napoca, Romania; georgemuntean99@gmail.com (G.-A.M.); stalu@umfcluj.ro (S.-D.N.); 2 Department of Anatomy, Faculty of Medicine and Pharmacy, University of Oradea, 1 Decembrie Street, 410068 Oradea, Romania; lariholhos@gmail.com; 3 Clinic of Ophthalmology, Emergency County Hospital, 3–5 Clinicilor Street, 400006 Cluj-Napoca, Romania

**Keywords:** vitelliform lesions, optical coherence tomography, image analysis, ImageJ, anti-VEGF

## Abstract

Acquired vitelliform lesions (AVLs) are associated with a large spectrum of retinal diseases, among which is age-related macular degeneration (AMD). The purpose of this study was to characterize AVLs’ evolution in AMD patients using optical coherence tomography (OCT) technology and ImageJ software. We measured AVLs’ size and density and followed their impacts over surrounding retinal layers. Average retinal pigment epithelium (RPE) thickness in the central 1 mm quadrant (45.89 ± 27.84 µm vs. 15.57 ± 1.40 µm) was significantly increased, as opposed to the outer nuclear layer (ONL) thickness, which was decreased (77.94 ± 18.30 µm vs. 88.64 ± 7.65 µm) in the vitelliform group compared to the control group. We found a continuous external limiting membrane (ELM) in 55.5% of the eyes compared to a continuous ellipsoid zone (EZ) in 22.2% of the eyes in the vitelliform group. The difference between the mean AVLs’ volume at baseline compared to the last visit for the nine eyes with ophthalmologic follow-up was not statistically significant (*p* = 0.725). The median follow-up duration was 11 months (range 5–56 months). Seven eyes (43.75%) were treated with intravitreal anti-vascular endothelium growth factor (anti-VEGF) agent injections, in which we noted a 6.43 ± 9 letter decrease in the best-corrected visual acuity (BCVA). The increased RPE thickness could suggest hyperplasia contrary to the decreased ONL, which could mirror the impact of the vitelliform lesion on photoreceptors (PR). Eyes that received anti-VEGF injections did not show signs of improvement regarding BCVA.

## 1. Introduction

The vitelliform macular lesion is classically found in Best’s vitelliform dystrophy but similar lesions can be identified in adults as “acquired vitelliform lesions” (AVLs). Gass described in 1974 a “peculiar foveomacular dystrophy”, which he further named “foveomacular vitelliform dystrophy: adult type” [1]. Over time, several terms have been applied to disorders similar to Gass’ description, such as vitelliform macular lesion, pseudo-vitelliform macular dystrophy, vitelliform macular degeneration, vitelliform lesion in adults, pseudo-vitelliform macular degeneration or adult vitelliform macular detachment in patients with basal laminar drusen [2].

The vitelliform lesion represents an accumulation of lipofuscin, melanosomes, melanolipofuscin and outer segment debris in the subretinal space, between the photoreceptors (PR) layer and retinal pigment epithelium (RPE) [3]. Since AVLs are mainly composed of pigmented granules of RPE origin, it was speculated by some authors that they might denote a specific RPE stress response to an unclarified insult [3]. Other authors observed a lack of direct apposition between the PR outer segments and RPE, which could be responsible for a delayed phagocytosis of shedding the outer segment photoreceptor tips, leading to the accumulation of yellow subretinal vitelliform material [4]. A clinicopathological case series performed on 14 eyes demonstrated two sources for the vitelliform material: an internal one, from the PR outer segment discs and an external one, from the RPE [1]. Electron-microscopy-based studies quantified the deposit’s ultrastructure and found that the largest component was a flocculent accumulation of small heterogeneous shapes (>50%), the second component was defined as “other”, the third component was RPE granules, the fourth component was smooth spherical profiles with homogeneous interiors resembling lipid droplets and the fifth component was constituted of PR outer-segments-derived materials [5].

Vitelliform lesions encompass a wide clinical spectrum of macular pathology such as degenerative (age-related macular degeneration—AMD), dystrophic (adult-onset foveomacular dystrophy—AOFVD), paraneoplastic, toxic (deferoxamine toxicity) as well as vitreoretinal interface (epiretinal membrane—ERM, vitreomacular traction—VMT) disorders [3,6]. The association between AVLs and cuticular drusen or chronic central serous chorioretinopathy was also noted [6]. Juliano et al. named the degenerative vitelliform lesions present in the elderly acquired vitelliform macular degeneration (AVMD). It occurs in cases without a family history of the disease, compared to an autosomal dominant inheritance pattern that characterizes the dystrophic vitelliform cases [7].

The relationship between AMD and vitelliform lesions was first noted by Gass. When AVL is associated with AMD, beside drusen, patients could also display subretinal drusenoid deposits (SDDs), hyperreflective foci (HRF), subretinal fluid (SRF), pigment epithelial detachments (PEDs), or macular neovascularization (MNV) [5]. The natural course of AVLs could be further complicated by choroidal neovascularization or foveal atrophy [3]. Thus, a study was carried out to analyze AVLs’ evolution in AMD eyes and revealed that 12% of AVLs led to type 1 MNV and 44% led to atrophy [5]. Due to the fact that there seems to be a strong preference of AVL for sub foveal location, it has been suggested that there might be a relationship with cones and their supporting cells [5]. In AMD, similarly to Best’s disease and AVMD, lipofuscin accumulates in the lysosomal compartment of the RPE, which seems to be one of the first and most important steps in the disease etiology [8].

The presence of subretinal fluid (SRF) in AVMD sometimes puzzles the clinician who must decide if the patient has a dry AMD converted to wet AMD or SRF with a vitelliform lesion [7,8]. The accumulation of SRF associated with vitelliform lesions was explained by a mechanical displacement between the RPE and outer retinal layers, which consequently inhibits the RPE from pumping out liquified lipofuscin debris [7]. 

Clinically, AVL is found as a slightly elevated yellow lesion, measuring approximately one-third to one disc diameter (DD) in size, with pigmentations in the form of a spot, figure or ring [1]. In optical coherence tomography (OCT), AVLs are characterized as a distinct layer of hyperreflective material between the PR and an irregularly contoured RPE band [5]. Optical coherence tomography angiography (OCTA)-based studies highlighted that the accumulation of the vitelliform material displaces the capillary network, inducing a decreased density of blood vessels at the superficial and deep capillary plexuses as well as the choriocapillaris [9].

BEST1 gene encodes a basolateral membrane protein called bestrophin 1 specific to RPE cells. This protein acts as an anion channel, having an important role in the transepithelial electrical potential and intracellular Ca^2+^ signaling [10]. Although it is specific for Best’s disease, a small number of BEST1 variants have been identified in patients with AMD, such as Thr216Ile and Leu567 alterations, which were found in three out of 259 AMD patients’ (1.1%) eyes [11]. Another five different alterations were found in five (1.5%) of 321 AMD patients as shown by Lotery A.J. et al. [12]: three patients carried a missense change (Arg105Cys, Glu119Gln, Val275Ile), while two carried a heterozygous nonsense mutation (Lys149X). Given the low frequency of mutation found in AMD patients, the authors stated that these patients could have been misdiagnosed, being in fact BEST1 mutations that mimic AMD [12].

Regarding AVLs’ evolution, the following scenario was described: an expansion phase is common to all AVLs, which is followed by either a collapse to complete RPE and outer retinal atrophy (cRORA), stability or resorption [5]. After reaching maximum expansion, the lesions that collapsed were completely resorbed after 24 months. Moreover, stable or resorbing lesions seem to be characterized by slower phases of growth and contraction [5]. Previous authors observed AVL complicated with MNV especially in the collapsing phase in 12% of study eyes [5]. 

In cases of AVL and SRF, the current literature offers no consensus regarding the benefit of anti-vascular endothelial growth factor (anti-VEGF) injections. In the context of a diagnostic dilemma, some patients receive anti-VEGF injections being mistaken as having neovascular AMD as opposed to AVL. Previous authors suggested two options: either to monitor closely and to treat only if the SRF increases and the patient becomes symptomatic or to give an anti-VEGF injection and determine the response in two weeks [7]. At this point, if the fluid disappears, there is a high chance of choroidal neovascularization (CNV), but if the SRF remains stable or slightly decreased, it can be assumed that the patient has AVMD [7]. 

The main purpose of the present study was to describe and analyse AVMD in adult patients using quantitative and qualitative features. A secondary purpose was to evaluate AVMD’s evolution in time and its response to anti-VEGF therapy.

## 2. Materials and Methods

### 2.1. Study Design

We conducted a retrospective, single-center study that was approved by the Ethics Committee belonging to “Iuliu Hațieganu” University of Medicine and Pharmacy (IHUMP), Cluj-Napoca, Romania (no.273/19.09.2022). The study protocol complied with the rules of the Declaration of Helsinki. 

### 2.2. Study Sample

We reviewed the OCT images of all consecutive patients who were diagnosed with macular pathology between 2017 and 2021, in the setting of the Emergency County Hospital, Clinic of Ophthalmology, Cluj-Napoca. The following combination of keywords were used in the search process: macular dystrophy or macular degeneration. The following diagnoses were found: macular atrophy 2 patients, macular degeneration 19 patients, macular dystrophy 3 patients, AMD 702 patients and maculopathy 34 patients. We further selected only patients with vitelliform lesions. The SD-OCT diagnosis of vitelliform material was based on the presence of a homogeneous or non-homogenous hyperreflective material above the RPE band that could not be classified as drusen or pigment epithelial detachment (PED). Fifteen patients met this criterion. The clinical stage of the disease applied on OCT images was determined according to Best’s disease classification: a. pre-vitelliform stage; b. vitelliform or egg yolk stage; c. pseudohypopyon stage; d. vitelliruptive or scrambled egg stage; e. atrophic stage, f. cicatricial and choroidal neovascularization [13]. Further on, 13 patients were considered eligible; 2 patients were excluded because of the poor OCT scans. Finally, a total of 18 eyes belonging to 13 patients with adult vitelliform lesions were included in the study. Because of the lack of family history enquiry and genetic testing or electrooculograms, the term “acquired vitelliform lesion” (AVL), was used to describe the patients with AVMD for the entire study [14].

**Figure 1:**
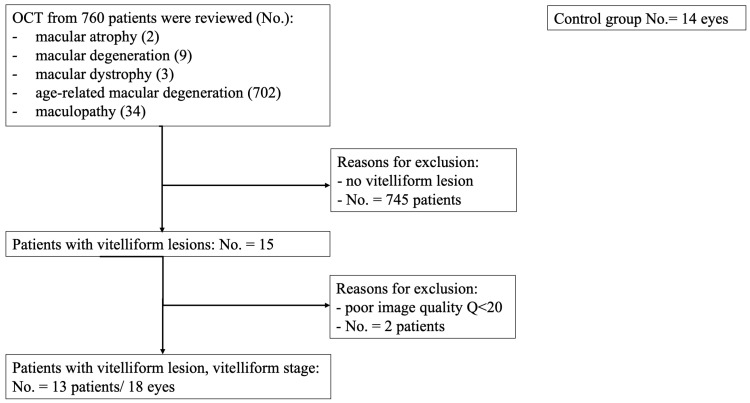
Flow diagram illustrating the study selection process. OCT = Optical coherence tomography; No = number.

Patients with an ophthalmologic examination in the same setting between January 2017 and September 2019, with no history of AMD, macular atrophy, macular degeneration or maculopathy were selected for the control group. Thus, we set up 2 groups of patients: vitelliform lesion (18 eyes) and control (14 eyes). The algorithm according to which the 2 groups were created is illustrated in Figure 1. 

Inclusion criteria for the vitelliform group were as follows:

1. Patients with a diagnosis of macular dystrophy or macular degeneration.
2. Minimal criterion for diagnosing AVLs was the presence of a macular round, hyperreflective lesion on the OCT, above the RPE.
3. Only high-quality (Q) OCT scans > 20 db (Q ranging from 0 = poor to 40 = excellent).

Exclusion criteria for vitelliform group:

1. Subretinal fibrosis, CNV, geographic atrophy (GA), epiretinal membrane, macular hole.
2. Eyes with poor-quality images preventing adequate grading Q < 20.

If a patient was confirmed with bilateral vitelliform lesion, both eyes were included in the study. 

Participants underwent VA testing with a Snellen visual acuity chart, slit lamp biomicroscopy and dilated eye fundus examination.

The following baseline clinical characteristics were recorded from the patients’ data files: age, gender, eye laterality, best-corrected visual acuity (BCVA), follow-up and type of intravitreal injection. Demographic data and ophthalmic examination were collected from the hospital’s informatic system. The OCT data were collected from the OCT database. 

Patients with at least 2 consecutive examinations and OCT images were further included for a detailed vitelliform lesion analysis, in which we studied the lesion’s evolution in time and we measured its volume.

### 2.3. Spectral Domain-OCT (SD-OCT) Data Aquisition

OCT was performed using a Spectralis OCT (Heidelberg Engineering, Inc. Heidelberg, Germany). All frames were reviewed for the presence of AVL by one of the authors (I.D.). The images were obtained after using the automated eye alignment eye tracking software (TruTrack< Heidelberg, Engineering). Two scanning protocols were used: 1. “Fast macular” scanning protocol: 25 horizontal raster lines per eye separated by 240 µm, centered on the fovea, with a 20 × 20° scan and automatic real mean value (ART value) set at 9, was used; or 2. “Posterior pole” scanning protocol: 61 horizontal raster lines per eye separated by 120 µm, centered on the fovea with a 30 × 25° scan and automatic real mean value (ART value) set at 9. Only images with more than a 20 db signal strength and with individual retinal layers that could be identified were used for the analysis. ETDRS 1 mm macular maps were used to report the macular thickness and the central 1 mm ring was defined as the central thickness.

Segmentation was automatically performed using the Spectralis software version 6.0. We defined the following parameters: 1. retinal pigment epithelium layer (RPE)—Bruch’s membrane complex—between the outer limit of the photoreceptor layer (PR1/2) and choriocapillaris’ inner boundary; 2. outer retina—between ELM and Bruch’s membrane but not including the latter; 3. outer nuclear layer (ONL)—between the outer plexiform layer (OPL) and ELM; 4. inner retina from the ILM to the external limiting membrane (ELM); 5. total retina thickness—between the internal limiting membrane (ILM). The inbuilt Spectralis mapping software was used to perform measurements from each SD-OCT scan. 

The images were reviewed by one of the authors (I.D.) before data analysis and manual adjustments to the retinal layer segmentation were made if necessary. 

In the OCT, the subretinal drusenoid deposit (SDD) was defined as a hyperreflective material above the RPE, between the RPE–Bruch membrane band and the ellipsoid zone (EZ) [5]. Soft drusen was defined as a deposit of material between the RPE–basal lamina (RPE–BL) and the inner collagenous layer of Bruch’s membrane [5]. Cuticular drusen was defined as RPE elevations in the form of blunted triangular apices, the bases of which sit on Bruch’s membrane with apices towards the neurosensory retina [5]. Subretinal fluid (SRF) appeared as a hyporeflective space located between the external limiting membrane (ELM) and the RPE–BL band [5]. Hyperreflective foci (HRF) were defined as solitary, small (<30 µm) or medium level hyperreflective retinal foci [15].

We described AVL’s fate as either: 1. collapsed—meaning a regressed AVL with cRORA, 2. stable AVL—meaning an AVL that did not completely regress during the follow-up, or 3. resorbed AVL, seen as a disappeared lesion with preservation of the surrounding tissue.

### 2.4. Vitelliform Lesions Analysis using Fiji Software

Images were analyzed using the public domain software, Fiji (version 1.53T, https://imagej.nih.gov/ij/ (accessed on 20 March 2022) [16]. Before uploading the OCT images to ImageJ, the vertical-to-horizontal ratio of the image was changed to 1:1 µm. We analyzed the non-stretched OCT scans to overcome the erroneous quantification of parameters [17]. First, the OCT image was opened in Fiji software and the scale was set. We measured a pixel length of 200 µm as given on the scale at the bottom of the OCT scan image, using the line tool. We further used the “magnifying glass” at 400% to obtain a better view of the vitelliform lesion and to easily identify its margins. With the help of the “polygon selection tool”, only the region with the vitelliform lesion was selected using the “crop button”. As previously defined by other authors, the anterior AVL boundary was considered the external limiting membrane, while the posterior AVL boundary was the retinal pigment epithelium–(basal lamina)–Bruch membrane (RPE–(BL)–BrM(RPE+BL–BrM)) complex [5]. If a PED was found, the posterior boundary was the RPE–BL. For each eye, we analyzed all the scans passing through the AVL and we chose the one passing through the fovea. On this particular scan, using the “straight line” option, we drew the base width and the maximum height of the vitelliform lesion. Using the “freehand line” tool we selected the entire vitelliform lesion to measure its perimeter. We performed 3 measurements for every parameter/eye and averaged them. The parameters are classified as follows:

#### 2.4.1. Vitelliform Lesion Size

**Figure 2:**
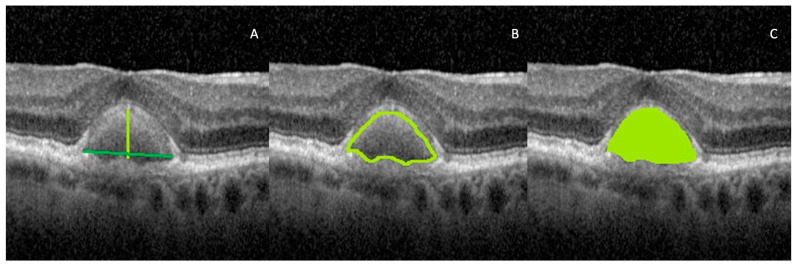
OCT images illustrating vitelliform lesion size parameters. (**A**) Maximum height (green light line) and base width (dark green line); (**B**) perimeter; (**C**) area. The stretched 1 × 1 pixel image was used for illustrative purposes only, to help visualize the details of the retinal structures.

- Maximum height: defined as the distance between the apex of the lesion and the RPE (see Figure 2. A: light green line). We defined apex as the vertex located at the highest point of the AVL or the point furthest from the base.
- Base width: defined as the distance between either edge of the lesion (see Figure 2A: dark green line).

Using the “freehand line” tool, the vitelliform lesion was delineated; this represents the region of interest (ROI) that was added to the ROI manager. If the AVLs were located on top of a drusenoid PED, we did not include the PED in the measurements. We set the needed measurements such as area and density characteristics, and then we pressed the measure button. We further extracted the results.

c Perimeter: defined as the total length of the vitelliform lesion outside the boundary (see Figure 2B).
d *Area* (*A*) (1): defined as the sum of the areas of each individual pixel, *a_p_*, in calibrated square units, within the borders of the lesion (see Figure 2C):

(1) $$A=\sum a_{p},\;$$

After the vitelliform lesion was delineated with the “freehand line” tool, we further analyzed various vitelliform lesion characteristics, as follows.

#### 2.4.2. Vitelliform Lesion Density

For the density parameters, we used the inbuilt Fiji measurements option.

- Mean gray value: defined as the sum of the gray values of all the pixels in the selection divided by the number of pixels.
- Modal gray value: defined as the most frequently occurring gray value within the selection. Corresponds to the highest peak in the histogram. Uses the heading mode.
- Min gray value: defined as the minimum gray value within the selection.
- Max gray value: defined as the maximum gray value within the selection.
- Integrated density (*IntDen*): defined as the product of area and mean gray value (2):

(2) IntDen=Area×Mean Gray value,

f Raw integrated density (RawIntdensity): defined as the sum of the values of the pixels in the vitelliform lesion.

#### 2.4.3. Vitelliform Lesions’ Volume and Evolution

**Figure 3:**
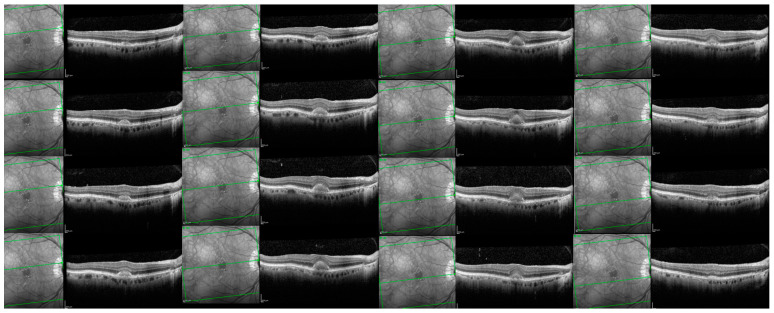
SD-OCT B-scans passing through the entire vitelliform lesion of one clinical study eye.

For 6 eyes with follow-up OCT images and an OCT Q over 20 dB, we collected all the B-scans that passed through the vitelliform lesions. Afterwards, we uploaded the images, one by one in Fiji, and calculated the area for each one of the segmentations (see Figure 3). In order to estimate the volume of the lesion, we summed up all the area measurements/eye and multiplied it with the distance between the sections (120 or 240 µm).

We compared the lesions’ size and density values from the first presentation with the follow-up. We also compared the thickness of the retinal layers from the vitelliform eyes with the normal eyes to find out if there was a connection between the vitelliform lesion’s presence and the surrounding layers. 

### 2.5. Statistical Analysis

The results are presented as percentages (%) for the categorical variables and mean ± standard deviation (SD) for the continuous variables. The normality of the data distribution was confirmed via the Kolmogorov–Smirnov test. We used the parametric Student’s *t*-test to compare the variables with a normal distribution between groups and non-parametric Mann–Whitney U tests for variables with a non-normal distribution. We used the *t*-test for the dependent means to compare the values from baseline to the last visit. In order to perform the statistical analysis, the Snellen Visual Acuity was converted into an approximate ETDRS letter score. Two-side, *p*-values < 0.05 were considered statistically significant. 

## 3. Results

### 3.1. General

More than 1500 SD-OCT images were analyzed by one of the authors (D.I.), followed by a more detailed analysis for 210 SD-OCT images performed by the same author. 

A total of 18 eyes belonging to 13 patients (4 (30.8%) females, 9 (69.2%) males) retrospectively diagnosed with AVLs were included in the study. The mean age at presentation was: 79.9 ± 7.1 years (range 67–91). Six patients (33.3%) had bilateral AVLs. The mean ETDRS score at baseline was 69 ± 18 letters.

**Figure 4:**
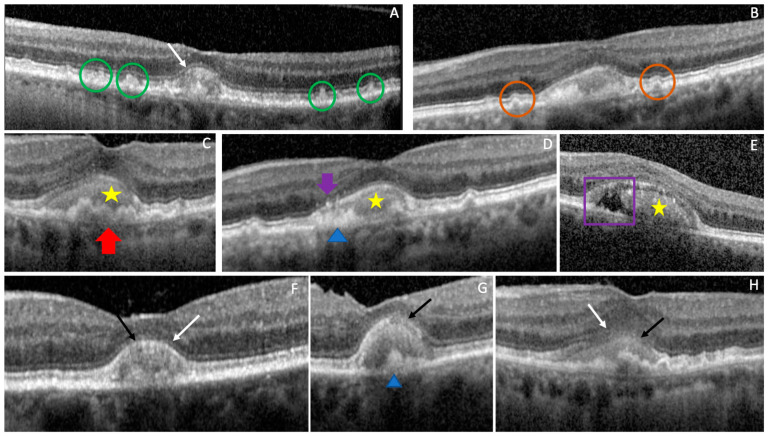
Associated retinal lesions. SD-OCT B-scans passing through the AVLs of clinical study eyes; (**A**) the green circles show SDD and the white arrow continuous ELM; (**B**) the brown circles show cuticular drusen; (**C**) the red arrow shows a drusenoid PED and the yellow star the vitelliform deposit; (**D**) the yellow star shows the vitelliform deposit, the purple arrow an HRF, the blue arrow head the RPE thickening; (**E**) the yellow star shows the vitelliform deposit, the purple square the SRF; (**F**) the white arrow indicates a continuous ELM and the black arrow a continuous EZ; (**G**): the black arrow indicates a discontinuous EZ and the blue arrow head the RPE thickening; (**H**) the white arrow indicates a indistinguishable ELM and the black arrow an indistinguishable EZ.

At baseline, 16/18 (88.9%) eyes presented a vitelliform stage while 2/18 (11.1%) presented a vitelliruptive stage. In terms of the OCT features found in our study eyes containing AVLs, 9 eyes (50%) had associated SDD, 8 eyes (44.4.%) had cuticular drusen, 4 eyes (22.2%) had soft drusen, 9 eyes (50%) had HRF, 5 eyes (27.8%) had SRF, 2 eyes (11.1%) had PED, and 3 eyes (16.6%) had no other findings, except the vitelliform lesion. The qualitative analysis showed a continuous ELM in 10/18 eyes (55.5%), a discontinuous ELM in 6/18 eyes (33.3%) and an indistinguishable ELM in 2/18 eyes (11.1%). EZ was continuous in 4/18 eyes (22.2%), discontinuous in 13/18 eyes (72.2%) and indistinguishable in 1/18 eyes (5.5%) (see Table 1 and Figure 4). 

Four patients/7 eyes (43.75%) received no follow-up. A total of 7 patients/9 eyes (56.25%) received an ophthalmological follow-up. The mean follow-up duration was: 23.33 ± 20.63 months (range 5–56 months). Six patients/six eyes with a well-defined AVL were further included in a specific volumetric analysis. 

Seven eyes were treated with anti-VEGF with a mean of 3 injections/eye (min 1–max 8). 

### 3.2. Vitelliform Lesions Analysis

At baseline, the average maximum height was 225.73 ± 336.33 µm (range 37.09–1211.60). The average base width size was 622.40 ± 419.71 µm (range 103.31–1855.37). The measured average perimeter was 1648.67 ± 943.90 µm (range 706.05–4274.17) while the average area was 17,803.28 ± 10,055.30 µm^2^ (range 7012.07–45,624.89) (See Table 2). The density of the lesions was further analyzed. The average mean gray value was 140.49 ± 16.54 (range 101.71–170.53), the average modal gray value was 130.37 ± 25.62 (range 78.86–165.22), the average min gray value was 65.30 ± 20.73 (range 20.29–104.24), the average maximum gray value was 212.74 ± 20.61 (range 175.43–248.78), the average integrated density was 2,366,898.95 ± 1,071,937 (range 892,257.21–4,640,455.74) and the average RawIntden was 20,614.39 ± 9630.70 (range 9034.45–40,785.26) (see Table 2). 

### 3.3. Retinal Layer Thickness

The different retinal layer thickness was assessed in the central 1mm subfield for the vitelliform and control groups (see supplementary Tables S1 and S2).

We compared the average values for the measured retinal layer thickness in the vitelliform and control groups. The RPE thickness in the central 1 mm (45.89 ± 27.84 µm vs. 15.57 ± 1.40 µm) as well as the RPE central max (101.67 ± 53.91 µm vs. 23.363.63 µm) were significantly increased in the vitelliform group compared to the control group (*p* < 0.001 and *p* < 0.001). The thickness in the central 1 mm (122.72 ± 36.09 µm vs. 86.43 ± 4.57 µm) and ORL central max (179.44 ± 56.83 µm vs. 97.36 ± 6.66 µm) were also significantly increased in the vitelliform group compared to the control (*p* < 0.001 and *p* < 0.001). ONL 1 mm was significantly decreased in the vitelliform group compared to the control: 77.94 ± 18.30 µm vs. 88.64 ± 7.65 µm (*p* = 0.049). The CRT in the central 1mm subfield (301.94 ± 50.02 µm vs. 258.43 ± 17.16 µm), the CRT min (252.94 ± 53.80 µm vs. 216.93 ± 19.87 µm) as well as the CRT max (345.19 ± 47.63 µm vs. 307.57 ± 16.10 µm) were significantly increased in the vitelliform group compared to the control (*p* = 0.005, *p* = 0.025 and *p* = 0.009) (see Table 3).

### 3.4. Vitelliform Lesions in Evolution

For six patients, a secondary analysis was carried out in order to investigate the evolution of the vitelliform lesions’ volume in time (see Table 4). The mean number of SD-OCT macular scans per eye used for the vitelliform volume plots was 9.22 ± 6.12 (range 3–25). In two eyes the volume increased while in four eyes it decreased. The difference between the mean vitelliform lesion’s volume at baseline and its volume at the last visit was not statistically significant (*p* = 0.725).

Further on, we calculated the difference between the baseline and the last visit in terms of vitelliform lesion size (see Table 5), density (see Supplementary Table S3), as well as RPE, ONL, ORL, IRL and CRT (see Table 6). The maximum height decreased in one eye while in five it increased (*p* = 0.232). The base width increased in three and decreased in three eyes (*p* = 0.125). The area and perimeter decreased in two eyes while in four it increased (*p* = 0.103 and *p* = 0.081).

The mean density of the lesion decreased in 3 eyes (*p* = 0.350), while the max gray level (*p* = 0.236) and the min gray level decreased in 4 eyes (*p* = 0.286). Eyes that had a decreased mean gray value presented decreased integrated density (*p* = 0.269) and RawIntdensity values (*p* = 0.232) (see Supplementary Table S3). 

Differences between the first and last presentation in the vitelliform group in terms of the retinal layer thickness are displayed in Table 6. The average RPE thickness, CRT and ORL for the 1 mm, min and max decreased at the last visit, but the differences were not significant (*p*> 0.05) (see Table 6).

At the last visit, 7/9 eyes (77.8%) presented with the vitelliform stage, while 2 of them (22.2%) presented with the vitelliruptive stage. The status of ELM was as follows: 3/9 (33.3%) continuous, 2/9 (22.2%) discontinuous and 4/9 eyes (44.4%) indistinguishable. EZ was discontinuous in 7/9 eyes (77.8%) and indistinguishable in 2/9 eyes (22.2%). In terms of the AVL’s fate, the vitelliform lesion increased in 7/9 eyes (77.8%), was stable with minimal change in 1/9 (11.1%) and in 1/9 eyes (11.1%) it had resorbed with a superior collapsed area. 

**Figure 5:**
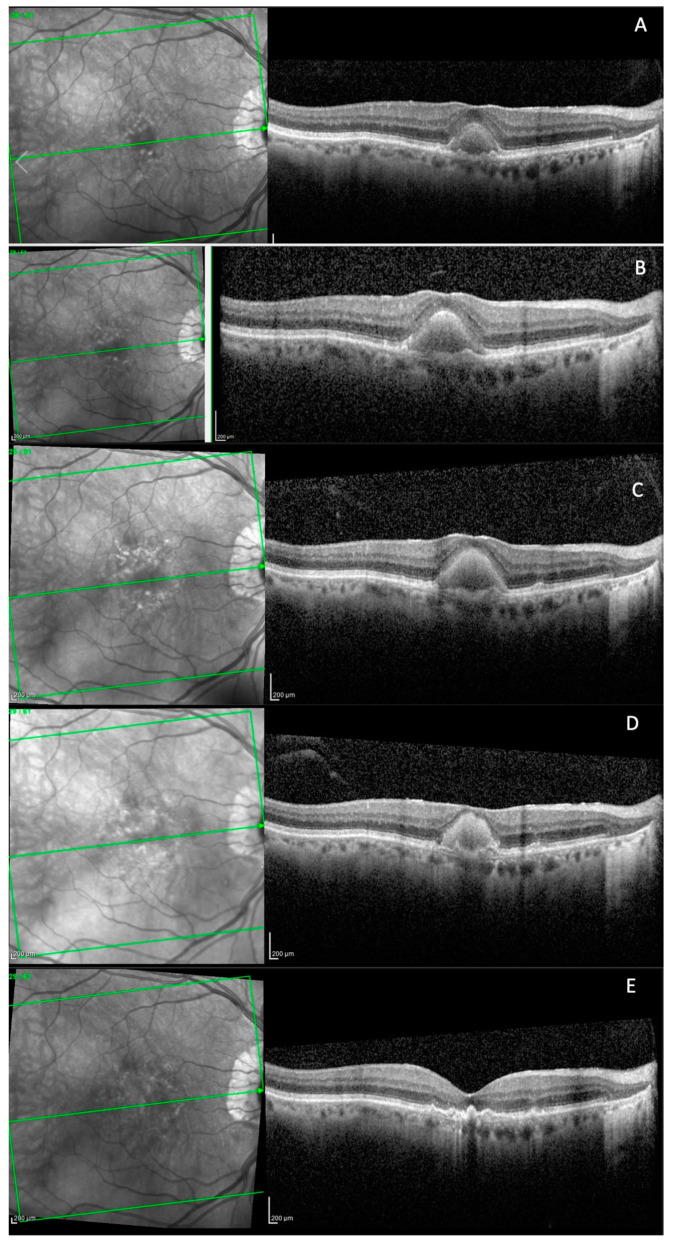
Spectral-domain OCT (SD-OCT) in a patient with AVL presenting the vitelliform stage at baseline (**A**); after 4 anti-VEGF injections with Bevacizumab, respectively 25 months later, the lesion increased (**B**); after another 2 anti-VEGF injections with Bevacizumab and 2 injections with Aflibercept (**C**), respectively, after another 14 months; after another 2 months the lesion started to shrink (**D**); after another month the lesion resorbed (**E**).

We further illustrated 2 representative cases. An 85-year-old patient, with a vitelliform lesion in the RE, with a BCVA of 80 ETDRS letters at baseline (see Figure 5A). After 25 months and 4 anti-VEGF Bevacizumab injections, the lesion increased in size (see Figure 5B). In the next 14 months, he received another 2 anti-VEGF injections with Bevacizumab and 2 anti-VEGF injections with Aflibercept (see Figure 5C). The lesion transformed into vitelliruptive while the BCVA was 70 ETDRS letters (see Figure 5E).

**Figure 6:**
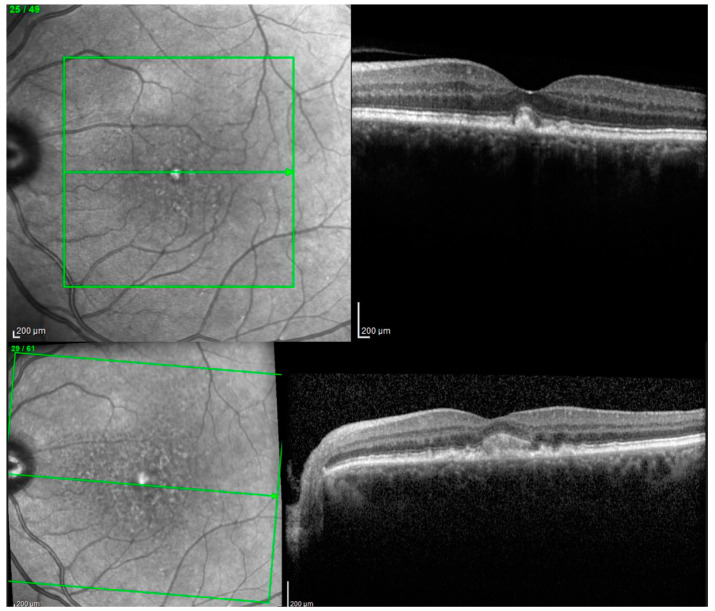
Spectral-domain OCT (SD-OCT) in patient with AVL presenting the vitelliform stage at baseline (**upper image**) and 43 months later at last visit (**lower image**).

Figure 6 presents the case of a 71-year-old patient with a small vitelliform lesion in his LE, with a BCVA ETDRS of 75 letters (A). Forty-three months later, the lesion increased, with a BCVA ETDRS of 75 letters (B).

Seven eyes (43.75%) were treated with intravitreal anti-VEGF agent injections. The initial and final BCVA is also provided along with the mean number of injections: 3/eye (min 1–max 8), and type of anti-VEGF agent: 16 Bevacizumab and 4 Aflibercept. Overall, the BCVA worsened between the baseline and final visits by −6.43 ± 9 letters, but the difference did not reach statistical significance (*p* = 0.054) (see Table 7). 

## 4. Discussion

In the present study, we used SD-OCT to characterize the anatomy of AVLs in terms of their dimension, density and relation with retinal layers. 

In our cohort, the AVLs were more frequent in patients in their seventh–eighth decade (79.25 ± 6.7 years). The age was similar to that reported in other studies 78.6 ± 6 years [18], as well as 72 years by Juliano J. et al. [7]. A slight male predominance was observed in our cohort 69.2% vs. 30.8% (*p* = 0.019), similar to the previously reported one: 56.25% vs. 43.75% [7]. 

In our study group, 33.3% had bilateral AVLs, similar to 31.25% found by Juliano J. et al. [7]. Saade C. et al. performed a review regarding AVLs in AMD patients and found 19% bilateral AVLs in a retrospective study with 19 eyes belonging to 16 patients, 44% bilateral AVLs in 13 eyes of 9 patients and 33% bilateral AVLs in 32 eyes of 24 patients [19].

We confirmed the presence of cuticular drusen in 10 eyes (62.5%), while others noted a prevalence of AVLs in patients with cuticular drusen from 1.2% to 24.2% [20]. The same author observed that the association between the AVL and cuticular drusen seems to lead to a collapse with the resolution of the vitelliform material, doubled by RPE failure and atrophy with the disappearance of drusen [20]. Contrary to another study in which all the 32 eyes belonging to 24 patients presented serous PED [21], we found only 2 eyes (12.5%) with RPE detachment. Hyperreflective foci (HRF) were present in 5 eyes (31.25%) in our series, while other authors found HRF in 9.8% of cases, of which five eyes also presented SRF (25%) [6]. 

Previous authors observed that ELM remains intact in most cases until complete AVL collapse and the subsequent development of the RPE and outer retinal atrophy [5]. Our study showed a continuous ELM in 55.5% compared to a continuous EZ in 22.2% of eyes. Based on our findings, it seems that the majority of eyes presented at baseline a discontinuous EZ and a continuous ELM. The discontinuity of the EZ could be explained by the findings from adaptive optics scanning laser ophthalmoscopy studies, which suggested that a rarefaction of cones doubled by morphological changes were found within the vitelliform lesion [22].

When analyzing the vitelliform lesion size, it seems that the base width was constantly larger compared to the maximum height. For both parameters, the range was large, which underlines the great variability between lesions: the average maximum height was 225.73 ± 335.33 µm (range 37.09–1211.60) and the average base width size was 622.40 ± 419.71 µm (range 103.31–1855.37). It was previously shown in an adjusted analysis that the baseline factors associated with an increased risk of MA included the base width (HR, 1.22; 95% CI, 1.16–1.28) and maximum height (HR, 2.61; 95% CI, 1.82–3.74) [23]. The perimeter and area analysis could further help in better describing the vitelliform lesions at the baseline and over time.

Similar values for the average and modal gray demonstrated a strong similarity between the density of the vitelliform lesions in our study group. One explanation could be the same disease stage, more precisely the vitelliform one. The average mean gray value was 140.49 ± 16.54 (range 101.71–170.53) and the average modal gray value was 130.37 ± 25.62 (range 78.86–165.22). It is noteworthy that to the best of our knowledge, this study is the first tentative study of density measurements for vitelliform lesions. This information helps to better describe changes within the lesion as it progresses. Moreover, it could represent a possible biomarker of disease progression. We also used a specific density marker called integrated density, which is related to the area in which the density was measured. On the same note, Boiko et al. investigated the retinal tissue area (RTA) and optical density (ODRT) of the retinal optical slice portion located in the central subfield, as well as their ratio (RTA/ODRT) in the presence of diabetic macular edema (DME) or of intraretinal cystic fluid (IRF) in neovascular age-related macular degeneration (nAMD) before and after anti-VEGF treatment [24]. The authors found that all the above-mentioned markers could be used as predictors of functional and anatomical outcomes since the RTA correlated positively both with post-anti-VEGF retinal thickness (r = 0.76; *p* < 0.001) and BCVA (r = 0.67; *p* < 0.001), while ODRT was moderately negatively correlated (r = 20.26; *p* = 0.049 and r = 20.48; *p* = 0.001, respectively) [24]. RTA/ODRT was strongly positively correlated (r = 0.75; *p* < 0.001 and r = 0.85; *p* < 0.001, respectively) [24]. 

The RPE, ORL thickness and CRT in the vitelliform group at baseline were increased compared to the control. This is contrary to other authors who found a similar RPE thickness with the control group, but they compared it with BVMD eyes [25]. As previously shown by other authors, the hyperreflective focal thickenings of the RPE band seen in SD-OCT may represent RPE hyperplasia or melanolipofuscin granules in the macrophages [14]. In terms of the RPE evolution, first it undergoes hypertrophy and then disruption, which could explain the increased RPE thickness values found in our study group. The ORL and CRT increase could mirror the RPE’s increased thickness. Agreeing with other descriptions, the central ONL thickness and min ONL thickness were decreased in our study, which could show the impact of the vitelliform lesion upon the PR and further on over the BCVA. As observed by another group, the ONL thickness was significantly lower compared to the control group and it correlated with the BCVA [26]. Other authors identified a photoreceptor equivalent thickness of 6.5 µm thicker in the BVMD compared to control [25]. The IRL 1mm and central max values were increased compared to the control group but were not statistically significant. Based on our findings, it seems that together with the RPE, the ORL layer and CRT are characterized by increased values. On the contrary, the ONL showed decreased values, as a proof of the vitelliform lesion impact on the PR.

All parameters analyzed in evolution have demonstrated increases as well as decreases in values, without a definite trend. We found a similar pattern for the density, retinal layer thickness, vertical height, horizontal diameter, area or perimeter, yet the changes were not significant. Previous authors suggested that during the disease progression, a change in the lesion’s composition or density might be possible, which further on could be responsible for the change in its overall reflectivity, from hyperreflective to hyporeflective [14]. Other authors found a significant shrinkage from 142.5 to 125.5 (*p* = 0.009) when comparing the initial versus final lesion height values in the AVL group [4]. However, from our data it was not possible to ascertain a significant decrease.

Data found by another study suggest that there might be an increase in the lesion’s size from the vitelliform stage to pseudohypopyon, while from the pseudohypopyon to the vitelliruptive stage there is a decrease in the lesion dimension, which could be a plausible explanation for our results [14]. In the same study, the authors found that during the disease evolution through different stages, when the lesion decreases, there is also a resorption of the vitelliform material doubled by a change in the lesion reflectivity: from hyperreflective to either mixed hyper + hyporeflective or hyporeflective, and also a photoreceptor loss [14]. The same authors raised the question about what triggers the fragmentation and resorption of the vitelliform material, which seems to remain unanswered [14]. 

At the final visit, the status of the ELM was discontinuous in 2/9 (22.2%) and indistinguishable in 4/9 eyes (44.4%), while the EZ was discontinuous in 7/9 eyes (77.8%) and indistinguishable in 2/9 eyes (22.2%). 

Regarding the AVL fate, most of the eyes demonstrated an increase in the vitelliform lesion (77.8%), 11.1% showed a stable lesion with minimal change and 11.1% progressed to resorption with a superior collapsed area.

When we analyzed the BCVA’s evolution in the eyes that received anti-VEGF agents, we found a negative trend but this was not statistically significant: −6.43 ± 9 letters (*p* = 0.054). Based on our findings, it seems that there is no benefit of anti-VEGF injections in eyes with vitelliform lesions, but larger studies need be performed in order to confirm this finding. These data are in accordance with those coming from other studies, which showed a significant mean BCVA reduction from the baseline to the last visit of at least 2 lines [2]. Another study underlined a visual acuity better or equal to 0.6 at presentation in 43% of the eyes that progressively decreased to 8% over 10 years of follow-up [27]. When the size of the lesion was analyzed in conjunction with the BCVA, a low correlation was found, so the authors speculated that BCVA seems to be less affected by quantitative changes (such as lesions size) and more dependent on qualitative changes such as the IS/OS interface integrity or reflectivity changes, since they found progression in the central photoreceptor IS/OS interface status and changes in the lesion reflectivity in the eyes with a significant mean BCVA reduction from baseline to the last visit [14]. These findings are in accordance with data from another study who found that the BCVA at baseline was best predicted by the IS/OS junction (*p* = 0.0002) and subfoveal integrity of the ELM (*p* = 0.001) in SD-OCT [28].

We acknowledge several limitations of our study. Firstly, the study is retrospective. Secondly, the sample size was small, limiting our ability to detect other statistical correlations. Thirdly, the genetic data to better differentiate between AOVMD, AVL and AMD are missing. Fourthly, the frequency of the follow-up between patients was variable. A complete automated method for vitelliform lesion identification and measurement would be useful in the future to decrease any potential biases from manual delineation.

## 5. Conclusions 

In our small series of AVLs in adult patients, we found increased RPE thickness as a sign of hyperplasia, contrary to the central and min ONL layer which were decreased, mirroring the impact of the vitelliform lesion over the PR. Most of the analyzed eyes presented a discontinuous EZ, both at baseline and in evolution, being a sign of visual impairment. During the follow-up some of the AVLs had increased while others regressed, which was translated in divergent results regarding height, width, area, perimeter or volume between the eyes. The density analysis helps us better evaluate the AVLs’ evolution in time, since vitelliform resorption is further translated into a reflectivity or density change. 

Eyes that received anti-VEGF injections did not show signs of improvement regarding the BCVA, since the mean change in our study group was −6.43 ± 9 letters (*p* = 0.054).

## Figures and Tables

**Table 1:** OCT features of patients with AVLs at baseline and last visit.

Stage of the Disease, n (%)	Baseline	Last Visit
Vitelliform	16/18 (88.9)	7/9 (77.8)
Pseudohypopyon	0/18 (0)	0/9 (0)
Vitelliruptive	2/18 (11.1)	2/9 (22.2)
Atrophic	0/18 (0)	0/9 (0)
SDD	9/18 (50)	4/9 (44.4)
Soft drusen	4/18 (22.2)	8/9 (88.8)
Cuticular drusen	8/18 (44.4)	4/9 (44.4)
SRF associated with AVL	5/18 (27.8)	0/9 (0)
HRF	9/18 (50)	3/9 (33.3)
PED	2/18 (11.1)	5/9 (55.5)
ELM		
Continuous	10/18 (55.5)	3/9 (33.3)
Discontinuous	6/18 (33.3)	2/9 (22.2)
Indistinguishable	2/18 (11.1)	4/9 (44.4)
EZ		
Continuous	4/18 (22.2)	0/9 (0)
Discontinuous	13/18 (72.2)	7/9 (77.8)
Indistinguishable	1/18 (5.5)	2/9 (22.2)

n = number; SDD = subretinal drusenoid deposit, SRF = subretinal fluid; AVL = acquired vitelliform lesion; HRF = hyperreflective foci; PED = pigment epithelium detachment; ELM = external limiting membrane; EZ = ellipsoid zone.

**Table 2:** Vitelliform lesion characteristics at baseline.

	Average ± SD	Median	Min	Max
Vitelliform Lesion Size				
Maximum height (µm)	225.73 ± 336.33	114.69	37.09	1211.60
Base width (µm)	622.40 ± 419.71	556.00	103.31	1855.37
Area (µm^2^)	17,803.28 ± 10,055.30	14,579.10	7012.07	45,624.89
Perimeter (µm)	1648.67 ± 943.90	1277.64	706.05	4274.17
Vitelliform lesion density				
Mean gray value	140.49 ± 16.54	143.39	101.71	170.53
Modal gray value	130.37 ± 25.62	132.54	78.86	165.22
Min gray level	65.30 ± 20.73	63.79	20.29	104.24
Max gray level	212.74 ± 20.61	211.64	175.43	248.78
Integrated density	2,366,898.95 ± 1,071,937.10	2,072,612.71	892,257.21	4,640,455.74
RawIntden	20,614.39 ± 9630.70	17,422.93	9034.45	40,785.26

SD = standard deviation; Min = minimum; Max = maximum; µm = micrometer. RawIntden = Raw integrated density.

**Table 3:** Comparing retinal layer thickness between vitelliform and control groups.

Average Retinal Layer Thickness (µm)	Vitelliform Group ± SD	Control Group ± SD	*p*
RPE 1 mm	45.89 ± 27.84	15.57 ± 1.40	<0.001
RPE central min	10.17 ± 5.37	10.71 ± 2.23	0.723
RPE central max	101.67 ± 53.91	23.36 ± 3.63	<0.001
ONL 1 mm	77.94 ± 18.30	88.64 ± 7.65	0.049
ONL central min	45.33 ± 20.34	53.86 ± 16.11	0.208
ONL central max	115.72 ± 21.84	112.14 ± 9.69	0.573
ORL 1 mm	122.72 ± 36.09	86.43 ± 4.57	<0.001
ORL central min	80.00 ± 11.08	78.14 ± 7.39	0.401 *
ORL central max	179.44 ± 56.83	97.36 ± 6.66	<0.001
IRL 1 mm	180.83 ± 30.89	172.14 ± 15.24	0.352
IRL central min	101.67 ± 40.35	121.36 ± 15.90	0.174
IRL central max	241.11 ± 35.81	216.29 ± 36.28	0.105
Retina 1 mm	303.00 ± 50.54	258.43 ± 17.16	0.004
Retina central min	254.17 ± 55.49	216.93 ± 19.87	0.023
Retina central max	348.17 ± 48.23	307.57 ± 16.10	0.005

* = Mann–Whitney U test. µm = micrometer; mm = millimeter; RPE = retinal pigment epithelium; min = minimum; max = maximum; ONL = outer nuclear layer; ORL = outer retinal layer; IRL = inner retinal layer, CRT = central retinal thickness. *p* indicates statistical significance.

**Table 4:** Vitelliform lesion volume.

Vitelliform Lesion Volume (µm^3^)			
Eye	Volume Baseline (µm^3^)	Volume Last Visit (µm^3^)	Difference 2–1 (µm^3^)
1	30,064,897.20	55,831,556.40	25,766,659.20
2	32,579,991.60	15,413,652.00	−17,166,339.60
3	4,310,901.60	20,337,099.60	16,026,198.00
4	13,922,006.40	9,493,209.60	−4,428,796.80
5	20,519,072.40	18,330,301.20	−2,188,771.20
6	17,674,183.20	17,100,080.40	−574,102.80
Average	19,845,175.40	22,750,983.20	2,905,807.80
Median	19,096,627.80	18,330,301.20	−574,102.80
Min	4,310,901.60	9,493,209.60	−17,166,339.60
Max	32,579,991.60	55,831,556.40	25,766,659.20

**Table 5:** Difference between baseline and last visit vitelliform lesion size.

	Maximum Height (µm)	Base Width (µm)	Area (µm^3^)	Perimeter (µm)
Average ± SD	−95.46 ± 296.04	248.27 ± 467.70	3398.72 ± 5733.13	327.48 ± 490.86
Median	9.51	21.68	1313.74	154.15
Min	−696.30	−83.26	−2509.03	−191.55
Max	87.32	1096.28	11,547.57	990.35

**Table 6:** Difference in retinal layer thickness between baseline and last visit in vitelliform group.

	Average ± SD	*p*
RPE 1 mm	−3.11 ± 22.87	0.347
RPE min	−2.67 ± 3.77	0.067
RPE max	−5.67 ± 61.03	0.393
ONL 1 mm	1.22 ± 15.67	0.821
ONL min	−5.56 ± 11.70	0.192
ONL max	22.56 ± 52.62	0.234
CRT 1mm	−14.44 ± 41.76	0.329
CRT min	−8.00 ± 48.73	0.635
CRT max	−18.33 ± 35.70	0.362
ORL 1 mm	−7.11 ± 60.87	0.379
ORL min	−6.22 ± 10.51	0.113
ORL max	−7.11 ± 60.87	0.735
IRL 1 mm	1.67 ± 15.48	0.754
IRL min	1.00 ± 30.19	0.923
IRL max	13.67 ± 43.99	0.378

µm = micrometer; mm = millimeter; RPE = retinal pigment epithelium; min = minimum; max = maximum; ONL = outer nuclear layer; ORL = outer retinal layer; IRL = inner retinal layer, CRT = central retinal thickness.

**Table 7:** Eyes with vitelliform lesions treated with anti-VEGF injections.

No.	Initial BCVA (Letters)	Follow-Up (Months)	Anti-VEGF Injections (No.)	Final BCVA (Letters)	Difference BCVA (Letters)
1.	80	29	8 (6B + 2A)	70	−10
2.	80	29	5 B	55	−25
3.	70	6	2 B	65	−5
4.	80	56	1A	80	0
5.	75	56	1 B	75	0
6.	85	5	2 B	80	−5
7.	25	11	1 A	25	0
average ± SD	70 ± 20.7	27.4 ± 21.87	2.9 ± 2.67	64.3 ± 19.46	−6.43 ± 9

BCVA = Best corrected visual acuity; No. = number; anti-VEGF = anti-vascular endothelial growth-factor; B = Bevacizumab; A = Aflibercept.

## Data Availability

The data presented in this study are available upon request from the corresponding author. The data are not publicly available due to privacy reasons.

## Supplementary Materials

The following supporting information can be downloaded at: https://www.mdpi.com/article/10.3390/biomedicines11051382/s1, Table S1: Retinal layer thickness in vitelliform group; Table S2: Retinal layer thickness in control group; Table S3: Difference between baseline and last visit vitelliform lesion density.
