# Supplementary material for: Advanced ImageJ Analysis in Degenerative Acquired Vitelliform Lesions Using Techniques Based on Optical Coherence Tomography
Source: Biomedicines. 2023 May 6;11(5):1382. doi: 10.3390/biomedicines11051382 (PMC10216572; doi:10.3390/biomedicines11051382)
Supplement: Supplementary file 1 [file biomedicines-11-01382-s001.zip › biomedicines-2349618-supplementary.pdf]

Table S1. Retinal layer thickness in vitelliform group.

**Retinal layer thickness (µm)**	Average	SD	Median	Min	Max
RPE 1 mm	45.89	27.84	34.50	15.00	101.00
RPE central min	10.17	5.37	10.50	1.00	25.00
RPE central max	101.67	53.91	96.00	32.00	199.00
ONL 1 mm	77.94	18.30	80.00	38.00	108.00
ONL central min	45.33	20.34	42.50	14.00	78.00
ONL central max	115.72	21.84	114.00	70.00	175.00
ORL 1 mm	122.72	36.09	109.00	81.00	178.00
ORL central min	80.0	11.08	77.50	72.00	116.00
ORL central max	179.44	56.83	175.00	93.00	252.00
IRL 1 mm	180.83	30.89	181.00	124.00	243.00
IRL central min	101.67	40.35	106.00	39.00	207.00
IRL central max	241.11	35.81	235.50	173.00	306.00
CRT 1 mm	303.00	50.54	288.00	234.00	415.00
CRT min	254.17	55.49	233.00	198.00	383.00
CRT max	348.17	48.23	343.50	281.00	441.00

µm= micrometer; mm= millimeter; RPE= retinal pigment epithelium; min= minimum; max=maximum; ONL= outer nuclear layer; ORL=outer retinal layer; IRL= inner retinal layer, CRT=central retinal thickness.

Table S2. Retinal layer thickness in control group

**Retinal layer thickness (µm)**	Average	SD	Median	Min	Max
RPE 1 mm	15.57	1.40	15.50	13.00	18.00
RPE central min	10.71	2.23	11.00	5.00	13.00
RPE central max	23.36	3.63	22.00	18.00	29.00
ONL 1 mm	88.64	7.65	86.00	77.00	102.00
ONL central min	53.86	16.11	59.00	34.00	85.00
ONL central max	112.14	9.69	111.00	99.00	138.00
ORL 1 mm	86.43	4.57	88.00	78.00	93.00
ORL central min	78.14	7.39	79.00	58.00	85.00
ORL central max	97.36	6.66	94.50	91.00	112.00
IRL 1 mm	172.14	15.24	173.00	151.00	197.00
IRL central min	121.36	15.90	120.00	90.00	151.00
IRL central max	216.29	36.28	224.50	98.00	242.00
CRT 1 mm	258.43	17.16	256.50	231.00	287.00
CRT min	216.93	19.87	214.50	180.00	257.00
CRT max	307.57	16.10	309.00	277.00	328.00

µm= micrometer; mm= millimeter; RPE= retinal pigment epithelium; min= minimum; max=maximum; ONL= outer nuclear layer; ORL=outer retinal layer; IRL= inner retinal layer, CRT=central retinal thickness.

Table S3. Difference between baseline and last visit vitelliform lesion density

Eye	Mean gray value	Modal gray value	Min gray level	Max gray level	Integrated density	RawIntden
1	1.58	35.15	-22.32	27.10	1146987.18	9882.15
2	-7.24	-7.96	-21.42	1.61	-239330.57	-1044.75
3	103.69	39.09	26.27	-0.55	1734356.01	12803.27
4	5.97	-39.42	21.65	-0.24	427745.25	4630.19
5	-0.49	12.89	-18.22	-21.45	-397584.95	-2670.26
6	-52.39	-48.97	-20.04	-67.54	-1013374.09	-8114.00
Average	8.52	-1.54	-5.68	-10.18	276466.47	2581.10
SD	51.29	37.23	23.05	32.05	1028612.08	7963.74
Median	0.55	2.47	-19.13	-0.40	94207.34	1792.72
Min	-52.39	-48.97	-22.32	-67.54	-1013374.09	-8114.00
Max	103.69	39.09	26.27	27.10	1734356.01	12803.27
